# Overexpression of SIRT1 in Mouse Forebrain Impairs Lipid/Glucose Metabolism and Motor Function

**DOI:** 10.1371/journal.pone.0021759

**Published:** 2011-06-30

**Authors:** Dongmei Wu, Yifu Qiu, Xiang Gao, Xiao-Bing Yuan, Qiwei Zhai

**Affiliations:** 1 Key Laboratory of Nutrition and Metabolism, Institute for Nutritional Sciences, Shanghai Institutes for Biological Sciences, Chinese Academy of Sciences, Graduate School of the Chinese Academy of Sciences, Shanghai, China; 2 Model Animal Research Center of Nanjing University, Nanjing, China; 3 Institute of Neuroscience and State Key Laboratory of Neurobiology, Shanghai Institutes for Biological Sciences, Chinese Academy of Sciences, Shanghai, China; Ecole Normale Supérieure de Lyon, France

## Abstract

SIRT1 plays crucial roles in glucose and lipid metabolism, and has various functions in different tissues including brain. The brain-specific SIRT1 knockout mice display defects in somatotropic signaling, memory and synaptic plasticity. And the female mice without SIRT1 in POMC neuron are more sensitive to diet-induced obesity. Here we created transgenic mice overexpressing SIRT1 in striatum and hippocampus under the control of CaMKIIα promoter. These mice, especially females, exhibited increased fat accumulation accompanied by significant upregulation of adipogenic genes in white adipose tissue. Glucose tolerance of the mice was also impaired with decreased Glut4 mRNA levels in muscle. Moreover, the SIRT1 overexpressing mice showed decreased energy expenditure, and concomitantly mitochondria-related genes were decreased in muscle. In addition, these mice showed unusual spontaneous physical activity pattern, decreased activity in open field and rotarod performance. Further studies demonstrated that SIRT1 deacetylated IRS-2, and upregulated phosphorylation level of IRS-2 and ERK1/2 in striatum. Meanwhile, the neurotransmitter signaling in striatum and the expression of endocrine hormones in hypothalamus and serum T3, T4 levels were altered. Taken together, our findings demonstrate that SIRT1 in forebrain regulates lipid/glucose metabolism and motor function.

## Introduction

Obesity is becoming a worldwide prevalent disease in recent years. Various diseases including diabetes, hepatic steatosis and atherosclerosis are associated with dysregulation of lipid metabolism. SIRT1, a NAD-dependant deacetylase, has been reported as a key regulator of energy homeostasis to be involved in lipid and glucose metabolism [1], [2], [3]. SIRT1 has been reported to attenuate adipogenesis and promote fat mobilization in adipocytes [4], [5]. SIRT1 liver-specific knockout mice showed better glucose tolerance and less fat accumulation in white adipose tissue (WAT) and liver than wild type when fed high-fat diet [6]. Deletion of SIRT1 in hepatocytes impaired PPARα signaling and decreased fatty acid β-oxidation [7]. SIRT1 induced hepatic glucose output through deacetylating PGC-1α in an NAD+-dependent manner [8]. Furthermore, our previous study showed that SIRT1 and resveratrol improved insulin sensitivity by repressing PTP1B transcription in hepatocytes or C2C12 myotubes [9]. SIRT1 also deacetylated PGC-1α and upregulated mitochondrial genes and fatty acid oxidation genes in skeletal muscle cells [10]. Moreover, pancreatic β cell-specific SIRT1 overexpressing transgenic mice exhibited improved glucose tolerance and enhanced insulin secretion in response to glucose stimulation [11]. And further study showed that SIRT1 repressed the uncoupling protein 2 transcription and positively regulated insulin secretion in pancreatic β cells [12]. Oral administration with SIRT1 activator resveratrol or SRT1720 in high-calorie-diet fed mice or diabetic mice improved hepatic, adipose and systemic insulin sensitivity, prevented the development of fatty liver and/or increased mitochondrial activity in the brown adipose tissue (BAT) and muscle [13], [14], [15]. All these studies demonstrate that SIRT1 plays crucial roles in lipid and glucose metabolism in peripheral tissues.

SIRT1 also plays important roles in central nervous system [16]. Overexpression of SIRT1 in APPswe/PSEN1dE9 mice alleviated brain pathology and behavioral deficits, and SIRT1 brain-specific knockout in the mice aggravated the symptoms [17]. Overexpression of SIRT1 in the CA1 region of p25 transgenic mice by injection of lentivirus also protected against neurodegeneration [18]. In addition, inhibition of SIRT1 in neurons increased IRS-2 acetylation and decreased phosphorylation of IRS-2 and ERK1/2 to protect neurons against oxidative stress [19]. Recently, brain-specific SIRT1 knockout mice showed defects in somatotropic signaling when fed ad libitum and defects in endocrine and behavioral responses under calorie restriction condition [20]. Knockout of SIRT1 in pro-opiomelanocortin (POMC) neurons caused reduced energy expenditure in mice and then hypersensitivity to diet-induced obesity [21]. The mice overexpressing SIRT1 driven by prion (PrP) promoter showed enhanced neural activity in hypothalamic nuclei, higher body temperature and physical activity when diet restricted [22]. It was also reported that SIRT1 played essential roles in memory and synaptic plasticity [23], [24]. Despite the huge progress in the understanding of functional importance and molecular mechanisms of SIRT1, the functions of SIRT1 in specific brain regions need more intensive study.

Given the important roles of SIRT1 in central nervous system, we developed a SIRT1 transgenic mouse model under the control of CaMKIIα promoter and investigated the role of SIRT1 in forebrain. We show that the transgenic mice exhibited increased fat accumulation, impaired glucose tolerance and motor function. And these changes were coupled with altered IRS-2 and neurotransmitter signaling in striatum and impaired expression of genes regulating lipid and glucose metabolism in various tissues. Altogether, our results demonstrate that SIRT1 in forebrain is very important for regulating lipid/glucose metabolism and motor function.

## Results

### Generation of bitransgenic mice with forebrain-specific SIRT1 overexpression

**Figure 1:**
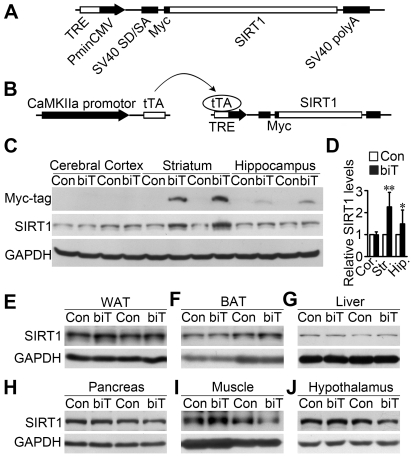
Generation of transgenic mice with forebrain-specific SIRT1 overexpression. (A) Schematic representation of the TRE-SIRT1 construct. (B) Genetic strategy to generate CaMKIIα-tTA/TRE-SIRT1 bitransgenic mice. (C) SIRT1 protein levels markedly increased in the striatum of bitransgenic mice (biT) compared with littermate controls (Con). Expression of SIRT1 in cerebral cortex (Cor.), striatum (Str.) and hippocampus (Hip.) were monitored by western blot using myc-tag or SIRT1 antibody. GAPDH was measured as loading control. (D) Quantification of the relative SIRT1 protein levels corresponding to (C). Except indicated, in this and all other figures, error bars represent SD. *n* = 4 pairs. * *P*<0.05, ** *P*<0.01 versus littermate controls. (E–J) SIRT1 protein levels were not changed in white adipose tissue (WAT) (E), brown adipose tissue (BAT) (F), liver (G), pancreas (H), muscle (I) and hypothalamus (J) as detected by western blot.

To further investigate the role of neuronal SIRT1, we first made the TRE-SIRT1 construct, which contains a tTA-responsive promoter followed by SIRT1 coding sequence with an N-terminal myc tag, to generate TRE-SIRT1 single transgenic mice (Fig. 1*A*). Then the single transgenic mice were crossed with CaMKIIα-tTA mice, which specifically drive tTA expression in forebrain [25], to create CaMKIIα-tTA/TRE-SIRT1 bitransgenic mice (Fig. 1*B*). Western blot analysis revealed that SIRT1 protein level was increased to 2.3-fold in striatum and 1.3-fold in hippocampus, but no obvious change in cerebral cortex of bitransgenic mice when compared with their littermate controls (Fig. 1*C and D*). Similar SIRT1 protein levels between bitransgenic mice and their littermates were detected in WAT, BAT, liver, pancreas, muscle and hypothalamus (Fig. 1*E*–*J*). These results demonstrate that the bitransgenic mice exhibit a forebrain-specific SIRT1 overexpression.

### Fat accumulation increases in the bitransgenic mice

**Figure 2:**
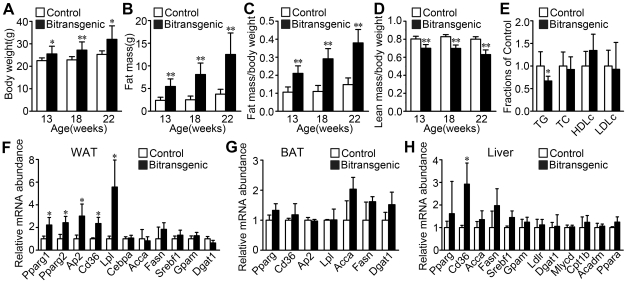
Female bitransgenic mice exhibit increased fat accumulation accompanied by increased expression of adipogenic genes in WAT. (A) Body weights of female bitransgenic mice increased when compared with littermate controls (*n* = 7–11 for each group). (B–C) Fat mass and fat content markedly increased in female bitransgenic mice compared with littermate controls (*n* = 7–11 for each group). (D) Lean content decreased in female bitransgenic mice compared with littermate controls (*n* = 7–11 for each group). (E) Fasting serum levels of total triglyceride (TG), cholesterol (TC), high-density lipoprotein cholesterol (HDLc), low-density lipoprotein cholesterol (LDLc) were measured at 3 months of age (*n* = 6–7 for each group). (F) The expression of some adipogenic genes increased in WAT of female bitransgenic mice. (G) The expression of the indicated adipogenic genes was not significantly changed in BAT of female bitransgenic mice. (H) The expression of the indicated adipogenic genes except Cd36 was not changed in liver of female bitransgenic mice. * *P*<0.05, ** *P*<0.01 versus littermate controls.

To explore the effect of forebrain SIRT1 on metabolism, we examined the body weight, fat and lean content of the bitransgenic mice. Interestingly, female bitransgenic mice showed progressive increases in body weight compared with littermate controls when measured at the age of 13, 18 or 22 weeks (Fig. 2*A*). Measurement of fat and lean mass using NMR showed that the fat mass and fat content of female bitransgenic mice was increased by two to three folds compared with littermate controls (Fig. 2*B and C*), while the lean content was slightly reduced (Fig. 2*D*). Therefore the increased body weight was mainly due to the dramatic increase of fat accumulation. Furthermore, we found that there was a decrease in fasting triglyceride level of female bitransgenic mice, while no significant changes were observed in the levels of total cholesterol, low-density lipoprotein cholesterol and high-density lipoprotein cholesterol (Fig. 2*E*).

PPARγ is a master regulator of adipogenesis [26], so we analyzed the mRNA levels of Pparg in WAT, BAT and liver. Pparg1 and 2 were both increased by two folds in WAT of female bitransgenic mice. Consistent with this result, the mRNA levels of PPARγ target genes including fatty acid binding protein 4 (Fabp4/Ap2), fatty acid transporter Cd36 and lipoprotein lipase (Lpl) were all increased markedly. And the mRNA levels of adipogenic gene Cebpa, *de novo* lipogenic genes Acca and Fasn, cholesterol synthesis gene Srebf1 and esterification genes Gpam and Dgat1 were not changed in WAT of female bitransgenic mice (Fig. 2*F*). Meanwhile, only significant increase of Cd36 was observed in liver (Fig. 2*H*), and no obvious difference was detected in BAT (Fig. 2*G*). Collectively, these results suggest that the upregulation of Pparg and its downstream genes in WAT probably accounts for the increase of fat mass in the female bitransgenic mice.

We also examined the body composition of male bitransgenic mice. They had similar body weight compared with their littermate controls (Fig. S1*A*). The fat mass and fat content of male bitransgenic mice also significantly increased as females (Fig. S1*B* and *C*), and the lean content was also slightly decreased when measured at the age of 18 or 22 weeks old (Fig. S1*D*). There were no significant changes of fasting triglyceride, total cholesterol, low-density lipoprotein cholesterol and high-density lipoprotein cholesterol levels in male bitransgenic mice (Fig. S1*E*). Taken together, these results show that forebrain-specific SIRT1 overexpression leads to the significant increase of fat content and the slight decrease of lean mass in both male and female bitransgenic mice.

### Forebrain-specific SIRT1 overexpression decreases glucose tolerance

**Figure 3:**
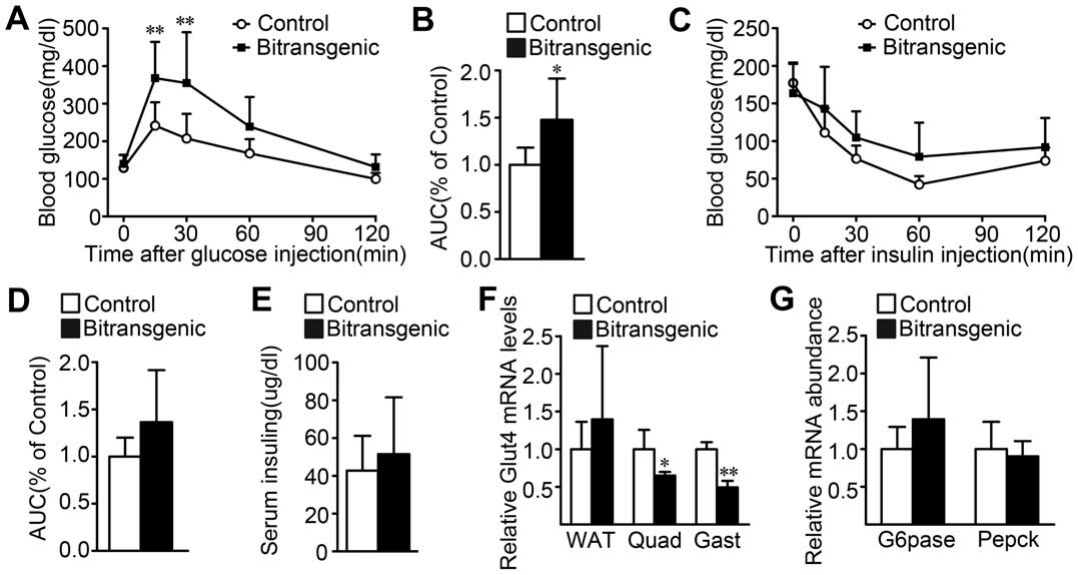
Forebrain-specific SIRT1 overexpression impairs glucose tolerance in female mice. (A) Glucose tolerance was impaired in 5-month-old female bitransgenic mice as determined by glucose tolerance test (*n* = 7 for each group). ** *P*<0.01 versus littermate controls by two-way ANOVA. Two-way ANOVA indicated that the curves for glucose tolerance were significantly different, *P* = 0.0199. (B) Forebrain-specific SIRT1 overexpression in females significantly increased the area under the curve (AUC) of the glucose tolerance test in (A). (C) 5-month-old female bitransgenic mice have similar insulin sensitivity as littermate controls when determined by insulin tolerance test (*n* = 6 for each group). (D) The AUC of the insulin tolerance test did not change in female bitransgenic mice. (E) Fasting serum insulin was measured at 3 months of age (*n* = 6–7 for each group). (F) The Glut4 mRNA levels in WAT, quadriceps (Quad) and gastrocnemius (Gast) muscle. (G) The G6pase and Pepck mRNA levels were comparable between bitransgenic mice and littermate controls. * *P*<0.05, ** *P*<0.01 versus littermate controls.

Obesity is usually associated with impaired glucose tolerance and insulin resistance [27], so we examined glucose tolerance and insulin sensitivity in the bitransgenic mice. Both female and male bitransgenic mice displayed impaired glucose tolerance when compared with their littermate controls (Figs. 3*A, 3B*, S2*A*, and S2*B*). Meanwhile, insulin tolerance test revealed that insulin sensitivity was not significantly altered in both female and male bitransgenic mice (Figs. 3*C, 3D*, S2*C* and S2*D*). In addition, the fasting serum insulin levels were not changed the bitransgenic mice (Figs. 3*E* and S2*E*). The two key enzymes of gluconeogenesis, G6pase and Pepck, remained unchanged in liver (Fig. 3*G*), while the Glut4 mRNA level was decreased about 50% in gastrocnemius and quadriceps, which might reduce the glucose uptake in muscle (Fig. 3*F*). These results demonstrate that the forebrain-specific SIRT1 overexpression leads to impaired glucose tolerance, which might partially due to the decreased Glut4 levels in muscle.

### Forebrain-specific SIRT1 overexpression decreases energy expenditure

**Figure 4:**
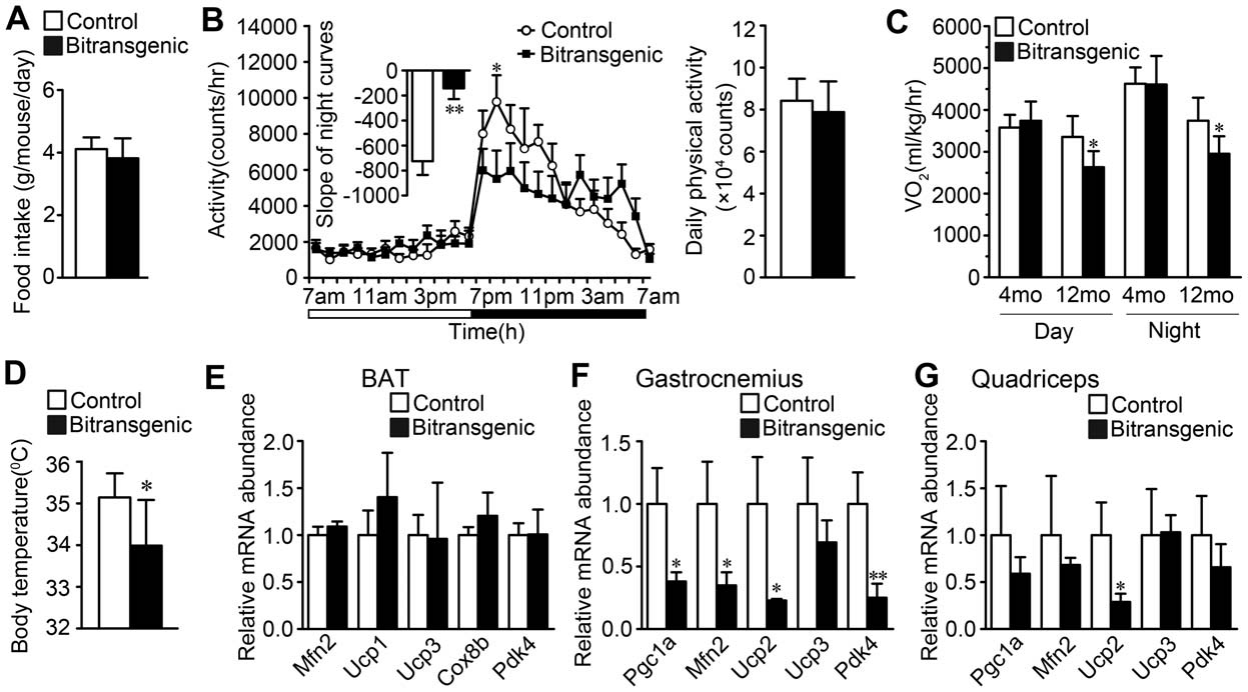
Female bitransgenic mice show abnormal spontaneous physical activity and decreased oxygen consumption and body temperature. (A) Food intake was similar between female bitransgenic mice and their littermate controls (*n* = 11 for each group). (B) Female bitransgenic mice had different physical activity pattern but similar daily physical activities when measured at 16 weeks of age through a 12-hour light/dark cycle using a comprehensive laboratory animal monitoring system (*n* = 7 for each group). Physical activity was presented as mean ± SEM. The slope coefficients of best-fit linear regression curves for the night activity were markedly different between bitransgenic mice and their littermate controls (shown as insert). (C) Oxygen consumption decreased in female bitransgenic mice at 12 months of age, *n* = 7 for each group at the age of 4-month old, *n* = 5 for each group at the age of 12-month old. (D) Body temperature decreased in female bitransgenic mice (*n* = 7 for each group). (E) The expression of the indicated mitochondrial genes did not change in the BAT of female bitransgenic mice when measured by real-time PCR. (F) The expression of some mitochondrial genes decreased in the gastrocnemius of female bitransgenic mice compared with littermate controls. (G) The expression of Ucp2 decreased in the quadriceps of female bitransgenic mice compared with littermate controls. * *P*<0.05, ** *P*<0.01 versus littermate controls.

We further investigated the effects of forebrain-specific SIRT1 overexpression on food intake, physical activity, oxygen consumption and body temperature. Female bitransgenic mice showed similar food intake as littermate controls, and male bitransgenic mice showed significantly decreased food intake when monitored from 13 to 18 weeks of age (Figs. 4*A* and S3*A*). Using a comprehensive laboratory animal monitoring system, female bitransgenic mice at four months of age showed similar daily physical activities but significantly reduced physical activities (by about 40%) between 8pm and 9pm and have a different spontaneous physical activity pattern at night (Fig. 4*B*). And the female bitransgenic mice displayed significantly lower oxygen consumption than their littermate controls at 12 months of age (Fig. 4*C*). In addition, the rectal temperature of the female bitransgenic mice were significantly decreased (Fig. 4*D*). Meanwhile, physical activity level, oxygen consumption and the rectal temperature of the male bitransgenic mice were similar as their littermate controls (Fig. S3*B*, *C* and *D*). These results demonstrate that the forebrain-specific SIRT1 overexpression leads to decreased energy expenditure which might contribute to the increased fat accumulation.

BAT and muscle are the most important organs responsible for regulating thermogenesis [28], so we examined the mitochondria-related genes in BAT, gastrocnemius and quadriceps. The genes including Pgc1a, Mfn2, Ucp2 and Pdk4 in gastrocnemius were markedly downregulated (Fig. 4*F*). The same downregulation was also observed with Ucp2 in quadriceps (Fig. 4*G*). However, these genes did not significantly change in BAT (Fig. 4*E*). The downregulation of mitochondria-related genes in gastrocnemius and quadriceps muscles might contribute to the diminished oxygen consumption and energy expenditure.

### Forebrain-specific SIRT1 overexpression impairs motor function

**Figure 5:**
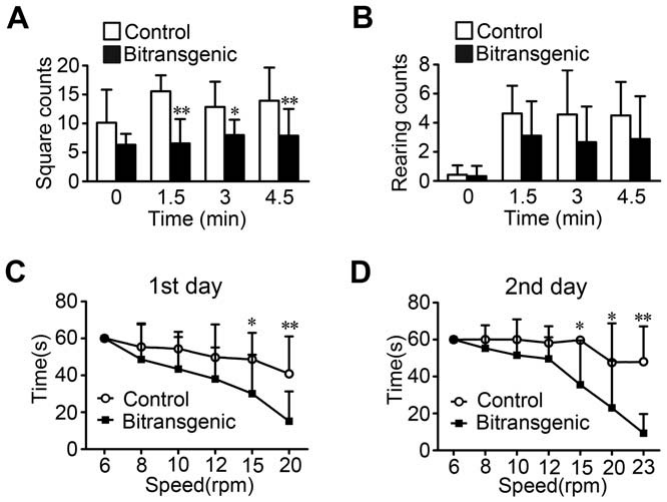
Female bitransgenic mice show decreased motor behavior by open field and rotarod tests. (A) Female bitransgenic mice crossed fewer squares in the open field test than littermate controls (*n* = 9–14 for each group). * *P*<0.05, ** *P*<0.01 versus littermate controls. Two-way ANOVA showed significant difference between groups, *P*<0.0001. (B) Female bitransgenic mice had similar number of rearings in the open field test (*n* = 9–14 for each group). (C) Rotarod performance on the first day was significantly decreased in female bitransgenic mice (*n* = 11 for each group). Mice were placed on a rod rotating for 60 seconds at the indicated speeds, and the latency to fall off from the rotarod was measured. * *P*<0.05, ** *P*<0.01 versus littermate controls by two-way ANOVA. Two-way ANOVA showed significant difference between curves, *P* = 0.0156. (D) Rotarod performance on the second day was significantly decreased in female bitransgenic mice (*n* = 11 for each group). * *P*<0.05, ** *P*<0.01 versus littermate controls by two-way ANOVA. Two-way ANOVA showed significant difference between curves, *P* = 0.0113.

Because striatum has a fundamental role in the control of motor activity [29], we evaluated the motor behavioral responses of the bitransgenic mice by open field test. Female bitransgenic mice crossed a lower number of squares than their littermate controls, while no significant change was observed in male bitransgenic mice (Figs. 5*A* and S4*A*). The number of rearings for both genders was similar to that of respective littermate controls (Figs. 5*B* and S4*B*). These data indicate that forebrain-specific SIRT1 overexpression in female mice impairs exploratory activity. When measured with rotarod test, female bitransgenic mice showed much shorter latency on rotarod than their littermate controls when the rotating speed reached 15 rpm on both the first and second days (Fig. 5*C* and *D*). The latency of male bitransgenic mice was also shorter than that of the control mice on both the first and second days (Fig. S4*C* and *D*). All these data indicate that forebrain-specific SIRT1 overexpressing mice develop impaired motor function. Meanwhile, improved performance was observed for both genotypes and both genders in the second day, which suggest that motor learning is normal in bitransgenic mice (Figs. 5*D* and S4*D*).

### SIRT1 overexpression alters IRS-2 and neurotransmitter signaling in striatum and expression of endocrine hormones in hypothalamus

**Figure 6:**
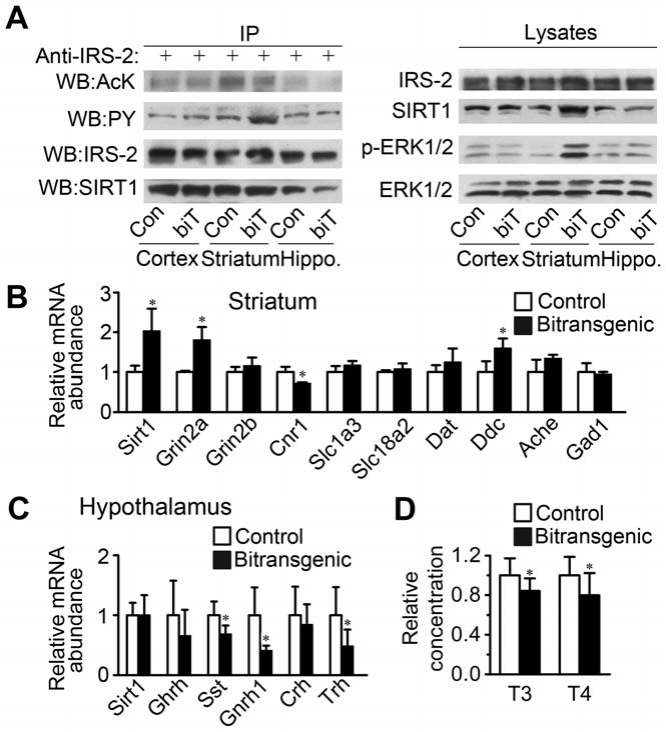
SIRT1 overexpression activates IRS-2 and ERK1/2 in striatum, and alters gene expression in striatum and hypothalamus. (A) Representative blots showed the effect of SIRT1 on acetylation of IRS-2 and phosphorylation of IRS-2 and ERK1/2 in the cerebral cortex, striatum and hippocampus of 3-month old female mice. The indicated tissues were lysed and immunoprecipitated with anti-IRS-2 and blotted with anti-acetylated-lysine (AcK), phosphotyrosine (PY), IRS-2 or SIRT1 antibody respectively. Lysates were also probed with IRS-2, SIRT1, phospho-ERK1/2 (p-ERK1/2) or ERK1/2 antibody respectively. (B) The expression of some genes involved in neurotransmitter signaling were altered in the striatum of female bitransgenic mice (*n* = 3 for each group). (C) Hypothalamic mRNA levels of some endocrine hormones in female bitransgenic mice were decreased when detected by real-time PCR (*n* = 6–7 for each group). * *P*<0.05 versus littermate controls. (D) Serum T3 and T4 levels in female bitransgenic mice were decreased when detected by ELISA (*n* = 10–11 for each group). * *P*<0.05 versus littermate controls.

It has been reported that knockdown of SIRT1 in cultured cortical neurons increased acetylation of IRS-2, and reduced phosphorylation of IRS-2 and ERK1/2 [19]. So we further examined these signaling events in forebrain of the female bitransgenic mice. Consistent with the previous in vitro results, SIRT1 was coimmunoprecipitated with IRS-2 in cerebral cortex, striatum and hippocampus (Fig. 6*A*). The SIRT1 overexpression in striatum decreased acetylation of IRS-2, and increased phosphorylation of IRS-2 and ERK1/2 (Fig. 6*A*). ERK1/2 signaling is essential for neuronal transcriptional regulation, and plays important roles in glutamate and dopamine signaling in striatum [30]. So we detected the expression of genes related to neurotransmitter signaling in striatum. Both NMDA receptor Grin2a and dopa decarboxylase Ddc increased in female bitransgenic mice. In addition, the mRNA level of cannabinoid receptor Cnr1, which regulates the release of neurotransmitters from axon terminals [31], was significantly decreased in female bitransgenic mice (Fig. 6*B*). Meanwhile, the mRNA level of SIRT1 in bitransgenic mice was markedly increased in striatum compared with their littermate controls.

PGC1α is an important substrate of SIRT1, and its deficiency in striatum is associated with certain behavioral abnormalities [8], [32]. In female bitransgenic mice, the mRNA levels of Pgc1a and its related genes in striatum were not changed when compared with their littermate controls (Fig. S6*C*).

It has been reported that SIRT1 improved insulin sensitivity by repressing PTP1B in C2C12 myotubes [9], and neuronal PTP1B regulated body weight, adiposity and leptin action in hypothalamus [33]. So we examined the mRNA and protein levels of PTP1B in the striatum and hypothalamus of female bitransgenic mice, and found they were not significantly changed (Fig. S5*A*–*C*). Meanwhile, the WAT leptin mRNA levels and fed serum leptin levels were elevated in female bitransgenic mice (Fig. S5*D*), suggesting that leptin might be involved in the forebrain SIRT1 function of metabolic regulation. Furthermore, leptin has been shown to stimulate Socs3 expression [34]. So we checked the mRNA levels of Lepr and Socs3 in the striatum and hypothalamus of female bitransgenic mice, and found that they were not altered (Fig. S5*E*). In addition, it has been shown that leptin regulated the expression of MCH, POMC, AgRP, NPY and CART in hypothalamus [35]. We found that the mRNA levels of neuropeptides including Pmch, Pomc, Agrp, Npy and Cartpt were not changed in the hypothalamus of female bitransgenic mice (Fig. S5*F*). These results are consistent with the normal food intake in the female bitransgenic mice (Fig. 4*A*). There were also normal SIRT1 mRNA levels in the hypothalamus of female bitransgenic mice, which is consistent with the normal SIRT1 protein levels (Fig. 1*J*). However, the mRNA levels of endocrine hormones including somatostatin, gonadotropin-releasing hormone and thyrotropin-releasing hormone were significantly decreased in the hypothalamus (Fig. 6*C*). Because hypothalamic somatostatin inhibits the release of growth hormone from the pituitary [36] and growth hormone regulates body length [37], we compared the snout-anus length between bitransgenic mice and controls. As shown in Fig. S6*B*, no significant difference was observed. The serum levels of thyroid hormones triiodothyronine (T3) and tetraiodothyronine (T4) in female and male bitransgenic mice were decreased compared to controls (Figs. 6*D* and S6*C*). The decreased expression of thyrotropin-releasing hormone in hypothalamus and reduced serum T3 and T4 levels might contribute to the changes in lipid and glucose metabolism of the bitransgenic mice.

## Discussion

In this study, we created CaMKIIα-tTA/TRE-SIRT1 bitransgenic mice which mainly overexpressed SIRT1 in striatum. The bitransgenic mice showed increased fat content, impaired glucose tolerance and decreased motor function. Further studies suggest that these phenotypes in female bitransgenic mice result from the altered IRS-2 and neurotransmitter signaling in striatum and impaired expression of genes regulating lipid and glucose metabolism in various tissues.

Our previous study showed that increased expression of SIRT1 can directly improve insulin sensitivity under insulin-resistant conditions in C2C12 myotubes [9]. The SIRT1 transgenic mice by knocking in SIRT1 cDNA into the β-actin locus became leaner, and displayed decreased fasting blood insulin and glucose levels and increased glucose tolerance, which was postulated due to SIRT1 overexpression in adipose tissue [38]. BAC-based transgenic mice overexpressing SIRT1 in various tissues exhibited normal fat mass and protected against high-fat diet-induced impaired glucose tolerance and hepatic steatosis due to decreased hepatic glucose production [39], [40]. These studies demonstrate that SIRT1 improves glucose and insulin tolerance in peripheral cells and tissues. SIRT1 POMC neuron-specific knockout female mice got more fat mass when fed on high-calorie diet [21]. Knockout of SIRT1 in Agrp neurons led to lower body weight, lean mass and fat mass in female mice [41]. Interestingly, our female bitransgenic mice overexpressing SIRT1 in forebrain, showed significant increase of fat mass (Fig. 2*B* and *C*). Furthermore, SIRT1 null mice displayed better glucose tolerance and hypermetabolic [12], [42]. SIRT1 brain-specific knockout mice displayed a reduction in fasting blood glucose level [20]. Consistently, our forebrain overexpressing SIRT1 mice showed impaired glucose tolerance (Figs. 3*A* and S2*A*) and decreased energy expenditure (Fig. 4*C*). In addition, we found that the SIRT1 mRNA levels were upregulated by fasting in hypothalamus and hippocampus, but not changed in striatum and other tested brain regions (Fig. S7). All the above findings suggest that SIRT1 may have different effects on glucose and lipid metabolism in different brain regions and peripheral tissues.

The male and female bitransgenic mice differed in the degree of fat accumulation at the same age (Figs. 2 and S1). The gender difference in the impairment of glucose tolerance might be related with the difference in the development of excess adiposity. The male mice even did not show obvious changes in open field and rotarod performance compared to female mice (Figs. 5 and S4). To investigate whether the overexpression levels of SIRT1 are contributed to the more obvious phenotype in female bitransgenic mice, we measured SIRT1 protein levels and found that the overexpression levels of SIRT1 are similar between the females and males in both striatum and hippocampus (data not shown). Thus, the more obvious phenotype in female bitransgenic mice should be mainly due to the gender difference, especially difference in gender-related hormones. The female bitransgenic mice with more fat accumulation might be related to the fact that females have a higher percentage of body fat, and adipocytes from female mice have increased lipogenic rates compared with those from males [43]. Although both male and female bitransgenic mice show similar alteration in T3 and T4 levels compared to control mice (Figs. 6*D* and S6*C*), the function and regulation of thyroid hormone depend on gender differently [44], [45]. In consistent with our observation, it has been reported that the mice with specific deletion of SIRT1 in POMC neurons show more pronounced changes of body weight in females than in males [21]. Similarly, the female mice with deletion of SIRT1 in Agrp neurons showed a more marked phenotype with reductions in fat mass than males [41]. Taken together, these studies indicate that the roles of SIRT1 in some distinct neurons may be significantly affected by gender.

The striatum is a major forebrain nucleus that has been proposed to play important roles in the development of motor deficits of Parkinson's disease and Huntington's disease [46]. The female bitransgenic mice with high expression of SIRT1 in striatum showed similar daily physical activity but reduced physical activity at the time point when normal mice reached peak activity (Fig. 4*B*), which is similar to the phenotype of mice treated with SIRT1 activator resveratrol [15]. It should be pointed out that the female bitransgenic mice maintained similar activity level throughout the nighttime without a peak of activity level as observed in the control mice (Fig. 4*B*). Both the SIRT1 transgenic mice by knocking in SIRT1 cDNA into the β-actin locus and the male mice treated with resveratrol showed improved rotarod performance [15], [38]. The bitransgenic mice overexpressing SIRT1 in forebrain showed impaired rotarod performance (Figs. 5*C, 5D*, S4*C* and S4*D*). The male mice treated with resveratrol or overexpressing SIRT1 under the control of rat neuron-specific enolase promoter, and the male SIRT1 null mice all exhibited normal activity during open field exploration [15], [23], [47]. Here, the male bitransgenic mice also showed similar behavior as their littermate controls in the open field test, but the female bitransgenic mice showed decreased crossed squares (Figs. S4*A* and 5*A*). These results show that SIRT1 in striatum or other tissues may regulate motor function, but the details need further investigation.

It has been shown that neuronal PTP1B regulated body weight, adiposity and leptin action [33], however the PTP1B expression was not altered in the striatum and hypothalamus of female bitransgenic mice (Fig. S5*A*–*C*). The WAT leptin mRNA levels and fed serum leptin levels were significantly increased in female bitransgenic mice (Fig. S5*D*). Meanwhile, the mRNA levels of Lepr and Socs3 in the striatum and hypothalamus, and the leptin-regulated neuropeptides including Pmch, Pomc, Agrp, Npy and Cartpt in hypothalamus were not altered in female bitransgenic mice (Fig. S5*E*). Combined with the normal food intake in the female bitransgenic mice (Fig. 4*A*), these data suggest that mild leptin resistant might be involved in the forebrain SIRT1 function of metabolic regulation. SIRT1 regulates systemic metabolism by deacetylating various proteins including PGC1α and IRS-2 [3]. We found that the expression levels of Pgc1a and its related genes did not alter in striatum (Fig. S6*A*), which suggests PGC1α is not a key substrate of SIRT1 in striatum. It has been reported that SIRT1 deacetylated IRS-2 in cultured cortical neurons, and the inhibition of SIRT1 impaired ERK1/2 activation [19]. Here we found that SIRT1 deacetylated IRS-2 and activated ERK1/2 signaling in striatum of female bitransgenic mice (Fig. 6*A*). ERK1-null mice were hyperactive in multiple motility tests including open field test [48], [49]. Therefore, we speculated that the increased phosphorylation of ERK1/2 might represent the underlying mechanisms of impaired motor function in open field and rotarod test for the bitransgenic mice. ERK1/2 mediated the NMDA, dopamine and endocannabinoid signalings, which have critical roles in the control of movement [46], [50], [51], [52]. So the increased expression of NMDA receptor Grin2a and dopa decarboxylase Ddc, and the decreased expression of endocannabinoid receptor Cnr1 in striatum might also contribute to the motor defects of the bitransgenic mice (Fig. 6*B*). However, the precise mechanisms by which SIRT1 regulate motor activity need to be further investigated. On the other hand, hypothalamus is very important in regulating metabolism. We detected the mRNA levels of endocrine hormones in hypothalamus, and found that the expression of somatostatin, gonadotropin-releasing hormone and thyrotropin-releasing hormone were changed in female bitransgenic mice (Fig. 6*C*). And the thyroid hormones T3 and T4 concentrations decreased in serum of bitransgenic mice (Figs. 6*D* and S6*C*). It was reported that thyroid hormones regulated adipocyte differentiation [53] and the transcription of genes such as UCPs [54]. Consistent with these reports, we found that some important adipogenic genes were markedly upregulated in WAT (Fig. 2*F*) and UCPs were decreased in muscle (Fig. 4*G* and *H*). It has been reported that hypothyroid mice showed decreased rotarod performance [55]. The rotarod performance of bitransgenic mice also decreased in our study (Fig. 5*C* and *D*). So the decreased hypothalamic thyrotropin-releasing hormone level and serum T3 and T4 levels might contribute to both the impaired lipid/glucose metabolism and the impaired motor function. The changes in hypothalamus may result from the alteration of the neurotransmitter signaling in striatum through striatal-hypothalamic circuitry [56]. These alterations in striatum and hypothalamus suggest a possible mechanism underlying the roles of forebrain SIRT1 in lipid/glucose metabolism and motor function. However, whether IRS-2 acetylation and tyrosine phosphorylation, ERK1/2 phosphorylation in striatum are essential for the impaired lipid/glucose metabolism and motor function in female bitransgenic mice needs to be studied in the future. Generation of mouse strains with forebrain specific point mutation of IRS-2 acetylation site or tyrosine phosphorylation site and forebrain specific deletion of ERK1/2 should be very helpful to elucidate the underlying mechanisms.

In conclusion, our study demonstrates overexpressing SIRT1 in mouse forebrain causes increased fat accumulation, impaired glucose tolerance and motor defects. These findings show that SIRT1 in different tissues may exert different impacts on lipid/glucose metabolism and motor function, which provide novel insights into the complexity and diversity of SIRT1 functions.

## Materials and Methods

### Generation of forebrain transgenic Mice

The generation of the TRE-SIRT1 mice using gene-targeting strategy was carried out at the Model Animal Research Center of Nanjing University (MARC). Briefly, the mouse SIRT1 cDNA from pBabe-SIRT1 plasmid (kindly provided by Dr. Shin-ichiro Imai, Washington University School of Medicine) was subcloned into pCMV-Myc vector (Clontech). And then the myc-SIRT1 fragment was introduced into pTRE2 vector (Clontech). After digested by ApaLI and XbaI, the fragment containing the tTA-responsive promoter followed by myc-SIRT1 was injected into mouse fertilized oocytes derived from C57BL/6×CBA F1 mice to generate TRE-SIRT1 transgenic mice. And then the TRE-SIRT1 transgenic mice were backcrossed for at least three generations with C57BL/6 mice obtained from Slaccas (Shanghai, China). The mice generated through the crossing of CaMKIIα-tTA mice [25] and TRE-SIRT1 mice were referred as bitransgenic mice, which were identified by PCR from their genomic DNA. The primers CAAAGGAGCAGATTAGTAAGCGG and TCCCCCTGAACCTGAAACATAAA were used to detect the exogenous SIRT1. The primers CGCTGTGGGGCATTTTACTTTAG and CATGTCCAGATCGAAATCGTC were used to detect tTA.

### Animal care

All animal experiments were performed in accordance with National Institutes of Health guidelines and with the approval of the Institutional Animal Care and Use Committee of the Institute for Nutritional Sciences. Mice were kept in specific pathogen-free conditions maintained at 22±3°C with a fixed 12-h light/dark cycle (lights on at 6:30 a.m.) with *ad libitum* access to standard chow and water in an institutional animal facility.

### Phenotypic analysis

In all the experiments, gender-matched littermates including CaMKIIα-tTA or TRE-SIRT1 single transgenic and wild type mice were used as controls. Mice were weighed at the indicated time points. The food intake was measured every 2 days from 13 weeks to 18 weeks of age. Rectal body temperatures of 8-month-old mice were measured using microprobe thermometer (Physitemp Instruments) between 14:00 and 16:00. Total body fat and lean mass were measured in conscious mice using a Minispec Mq7.5 Analyzer (Bruker). Mice were anesthetized with sodium pentobarbital, and then cerebral cortex, striatum, hippocampus, hypothalamus, liver, white adipose tissue, brown adipose tissue and muscle were quickly removed and snap-frozen in liquid nitrogen and stored at −80°C for western blot, immunoprecipitation and real-time PCR. For fasting and refeeding experiments, 8-week-old C57BL/6J female mice were fed *ad libitum*, fasted for 24 h, or fasted for 24 h and refed for 24 h (5–7 mice for each group). Then, animals were sacrificed, and the indicated brain regions were removed and snap-frozen for real-time PCR. Snout-anus length was measured with a micrometer on 12-week-old anaesthetized mice. Blood samples were obtained fed or after a 20-h fasting and stored at −80°C. Fed leptin level was measured using an ELISA kit from R&D systems. Serum insulin level was measured using a radioimmunoassay kit and T3, T4 levels were measured using ELISA kits from Beijing Beifang Institute of Biological Products. Triglyceride, total cholesterol, high-density lipoprotein cholesterol and low-density lipoprotein cholesterol levels in serum were determined using enzymatic assay kits from Shanghai Shensuo Unf Medical Diagnostics Articles Company.

### Glucose tolerance test and insulin tolerance test

Glucose and insulin tolerance tests were performed as previously described [9]. Briefly, after fasting for 12 h or 6 h, mice were injected intraperitoneally with either 2 g/kg of glucose or 0.75 U/kg of human insulin (Lilly France S.A.S) respectively. Tail blood glucose was measured before and 15, 30, 60 or 120 min after the injection using the FreeStyle blood glucose monitoring system (TheraSense).

### Oxygen consumption and physical activity

Oxygen consumption and physical activity were determined with mice fed *ad libitum* using a comprehensive laboratory animal monitoring system (Columbus Instruments) according to the manufacturer's instructions. Mice were measured for 24 h after acclimated to the system for 16 to 20 h.

### Open field test

The open field consisted of a box (50×30×20 cm) with normal room illumination, which was divided into 10×10 cm squares and open at the top. Each mouse at 12 weeks of age was placed in the centre of the box, and the number of squares entered (all four paws inside the square) and rearings were counted for 0.5 min for 4 times with an interval of 1 min for each time. The box was thoroughly cleaned to remove odor cues before each mouse had been tested.

### Rotarod test

The rotating rod was 3 cm in diameter and divided by flanges in five compartments to allow testing of up to five mice simultaneously [57]. The animals had to walk on the rotating rod at the indicated speeds, and the time until the mouse fell from the rod or maintained for 60 s was recorded. Mice at 8 months of age were tested from the lowest speed to gradually-increased speed, and were rested for about 40 min between each two tests with different speeds.

### Western blot and coimmunoprecipitation

IRS2, SIRT1 and phosphotyrosine antibodies were from Upstate; PTP1B antibody was from Novus Biologicals; acetylated-lysine (AcK), ERK1/2 and phospho-ERK1/2 (Thr202/Tyr204) antibodies were from Cell Signaling Technology; myc-tag antibody was kindly provided by Dr. J. Zhou (Institute of Biochemistry and Cell Biology, SIBS, CAS); GAPDH antibody was from Kangcheng (Shanghai, China). Western blot and coimmunoprecipitation was performed as previously described [58].

### Real-time PCR

Total RNA from the collected tissues was isolated using TRIzol reagent (Invitrogen). After treatment with RNase-free DNase I (Takara), first-strand cDNA was synthesized with reverse transcriptase and random hexamer primers (Invitrogen). Real-time PCR was conducted using Power SYBR Green PCR Master Mix with the ABI Prism 7900 sequence detection system (Applied Biosystems) as described previously [59]. The primers used for real-time PCR are mainly from PrimerBank (http://pga.mgh.harvard.edu/primerbank), and listed in Table S1. 36B4 was measured for each sample as the internal control. Three mice per group at 12 weeks of age were used in real-time PCR except indicated.

### Statistical analyses

Data are expressed as mean ± SD except indicated. Statistical analysis of differences was done via unpaired two-tailed Student's *t* test except indicated. Two-way analysis of variance (ANOVA) followed by Bonferroni's test was performed using GraphPad Prism 5.0 (GraphPad Software). *P*<0.05 was considered statistically significant.

## Supporting Information

Figure S1 **Male bitransgenic mice exhibit increased fat accumulation.** (A) Body weights of male bitransgenic mice were not changed compared with littermate controls (*n* = 6–8 for each group). (B–C) Fat mass and fat content increased in male bitransgenic mice when compared with littermate controls (*n* = 6–8 for each group). (D) Lean contents decreased in male bitransgenic mice when compared with littermate controls (*n* = 6–8 for each group). (E) Fasting serum levels of triglyceride (TG), total cholesterol (TC), high-density lipoprotein cholesterol (HDLc) and low-density lipoprotein cholesterol (LDLc) were measured at 3 months of age (*n* = 6–7 for each group). * *P*<0.05, ** *P*<0.01 versus littermate controls.
(TIF)

Figure S2 **Glucose tolerance is moderately impaired in male bitransgenic mice.** (A) Glucose tolerance was impaired in 5-month-old male bitransgenic mice as determined by glucose tolerance test (*n* = 6–7 for each group). Two-way ANOVA indicated that the curves for glucose tolerance are significantly different, *P* = 0.0376. (B) The area under the curve (AUC) of the glucose tolerance test in (A) was similar. (C) 5-month-old male transgenic mice have similar insulin sensitivity as determined by insulin tolerance test (*n* = 6–7 for each group). (D) The AUC of the insulin tolerance test in (C) did not change in male transgenic mice (*n* = 6–7 for each group). (E) Fasting serum insulin was measured at 3 months of age (*n* = 6–7 for each group). * *P*<0.05 versus littermate controls.
(TIF)

Figure S3 **Male bitransgenic mice show similar physical activity, oxygen consumption, body temperature and decreased food intake.** (A) Food intake of male bitransgenic mice decreased when compared with littermate controls (*n* = 5 for each group). (B) Physical activity did not change in male bitransgenic mice at 16 weeks of age, measured through a 12-h light/dark cycle (*n* = 5 for each group). Physical activity was presented as mean ± SEM. (C) Oxygen consumption did not change in male bitransgenic mice (*n* = 4–5 for each group). (D) Body temperature did not change in male bitransgenic mice at 8 months of age (*n* = 5 for each group). * *P*<0.05 versus littermate controls.
(TIF)

Figure S4 **Male bitransgenic mice show decreased motor behavior by open field and rotarod performance tests.** (A) Crossed squares in the open field test did not change in male bitransgenic mice (*n* = 5 for each group). Two-way ANOVA showed no significant difference between groups. (B) Male bitransgenic mice had similar number of rearings in the open field test (*n* = 5 for each group). Two-way ANOVA showed no significant difference between groups. (C) Rotarod performance on the first day was decreased in male bitransgenic mice (*n* = 5 for each group). ** *P*<0.01 versus littermate controls by two-way ANOVA. Two-way ANOVA showed significant difference between curves, *P* = 0.0225. (D) Rotarod performance on the second day was slightly decreased in male bitransgenic mice (*n* = 5 for each group). * *P*<0.05, ** *P*<0.01 versus littermate controls by two-way ANOVA.
(TIF)

Figure S5 **Leptin is upregulated in female bitransgenic mice, and the expression of PTP1B, Lepr, Socs3 and some feeding related neuropeptides is not changed in striatum or hypothalamus.** (A) The mRNA levels of PTP1B in the striatum (*n* = 3 for each group) and hypothalamus (*n* = 6–7 for each group) of female bitransgenic mice were not changed. (B) The protein levels of PTP1B in the striatum and hypothalamus of female bitransgenic mice were not changed (*n* = 3 for each group). GAPDH was measured as loading control. (C) Quantification of the relative PTP1B protein levels corresponding to (B). (D) The mRNA levels of leptin in WAT (*n* = 3 for each group) and fed serum leptin levels (*n* = 10 for each group) of female bitransgenic mice were significantly elevated. (E) The mRNA levels of Lepr, Socs3 in the striatum (*n* = 3 for each group) and hypothalamus (*n* = 6–7 for each group) of female bitransgenic mice were not changed. (F) The mRNA levels of some feeding related neuropeptides in hypothalamus of female bitransgenic mice were not changed (*n* = 6–7 for each group). * *P*<0.05, ** *P*<0.01 versus littermate controls.
(TIF)

Figure S6 **The mRNA levels of Pgc1a related genes in the striatum of female mice, the mouse snout-anus length and male serum T3, T4 levels.** (A) The expression of Pgc1a and its related genes did not alter in the striatum of female bitransgenic mice (*n* = 3 for each group). (B) The mouse snout-anus length was similar between bitransgenic mice and controls (*n* = 6–7 for each group). (C) Serum T3 and T4 levels were decreased in male bitransgenic mice (*n* = 10–11 for each group). * *P*<0.05 versus littermate controls.
(TIF)

Figure S7 **The Sirt1 mRNA levels in different brain regions under feeding, fasting and refeeding conditions.** The Sirt1 mRNA levels of olfactory bulb, cerebral cortex, striatum, hippocampus, hypothalamus, thalamus and hindbrain from 8-week-old female mice fed *ad libitum*, fasted for 24 h, or fasted for 24 h and refed for 24 h were analyzed by real-time PCR (*n* = 5–7). * *P*<0.05 versus fed *ad libitum*.
(TIF)

Table S1 **Primers used in real-time PCR.**
(DOC)
