# Supplementary figures and images for: Overexpression of SIRT1 in Mouse Forebrain Impairs Lipid/Glucose Metabolism and Motor Function
Source: PLoS One. 2011 Jun 30;6(6):e21759. doi: 10.1371/journal.pone.0021759 (PMC3128079; doi:10.1371/journal.pone.0021759)

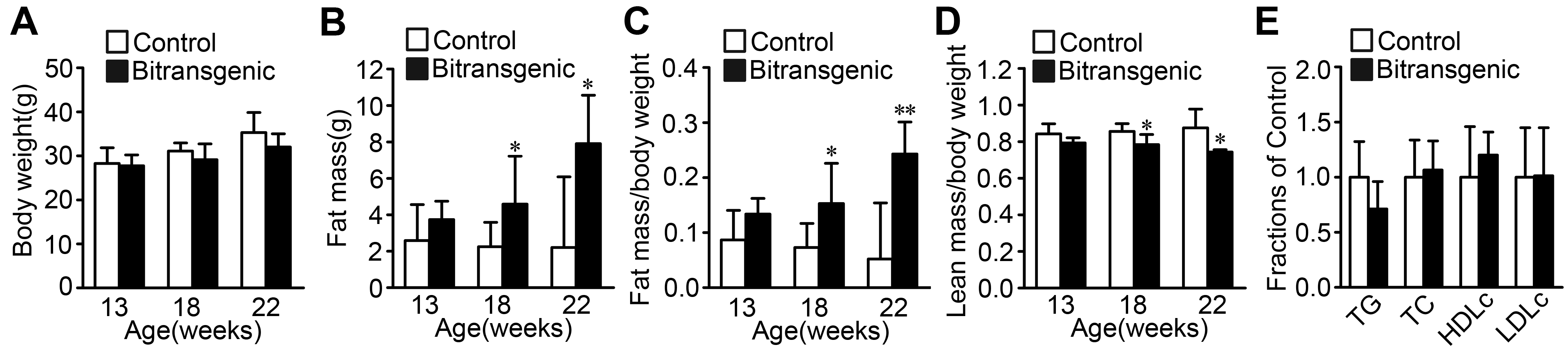

Supplement: Figure S1 — Male bitransgenic mice exhibit increased fat accumulation. (A) Body weights of male bitransgenic mice were not changed compared with littermate controls (n = 6–8 for each group). (B–C) Fat mass and fat content increased in male bitransgenic mice when compared with littermate controls (n = 6–8 for each group). (D) Lean contents decreased in male bitransgenic mice when compared with littermate controls (n = 6–8 for each group). (E) Fasting serum levels of triglyceride (TG), total cholesterol (TC), high-density lipoprotein cholesterol (HDLc) and low-density lipoprotein cholesterol (LDLc) were measured at 3 months of age (n = 6–7 for each group). * P<0.05, ** P<0.01 versus littermate controls. (TIF) [file pone.0021759.s001.tif]

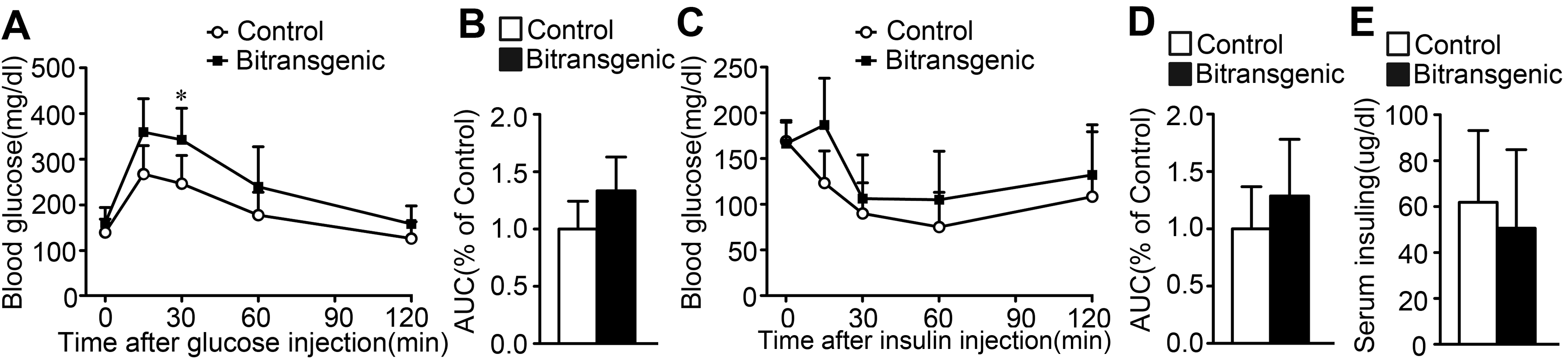

Supplement: Figure S2 — Glucose tolerance is moderately impaired in male bitransgenic mice. (A) Glucose tolerance was impaired in 5-month-old male bitransgenic mice as determined by glucose tolerance test (n = 6–7 for each group). Two-way ANOVA indicated that the curves for glucose tolerance are significantly different, P = 0.0376. (B) The area under the curve (AUC) of the glucose tolerance test in (A) was similar. (C) 5-month-old male transgenic mice have similar insulin sensitivity as determined by insulin tolerance test (n = 6–7 for each group). (D) The AUC of the insulin tolerance test in (C) did not change in male transgenic mice (n = 6–7 for each group). (E) Fasting serum insulin was measured at 3 months of age (n = 6–7 for each group). * P<0.05 versus littermate controls. (TIF) [file pone.0021759.s002.tif]

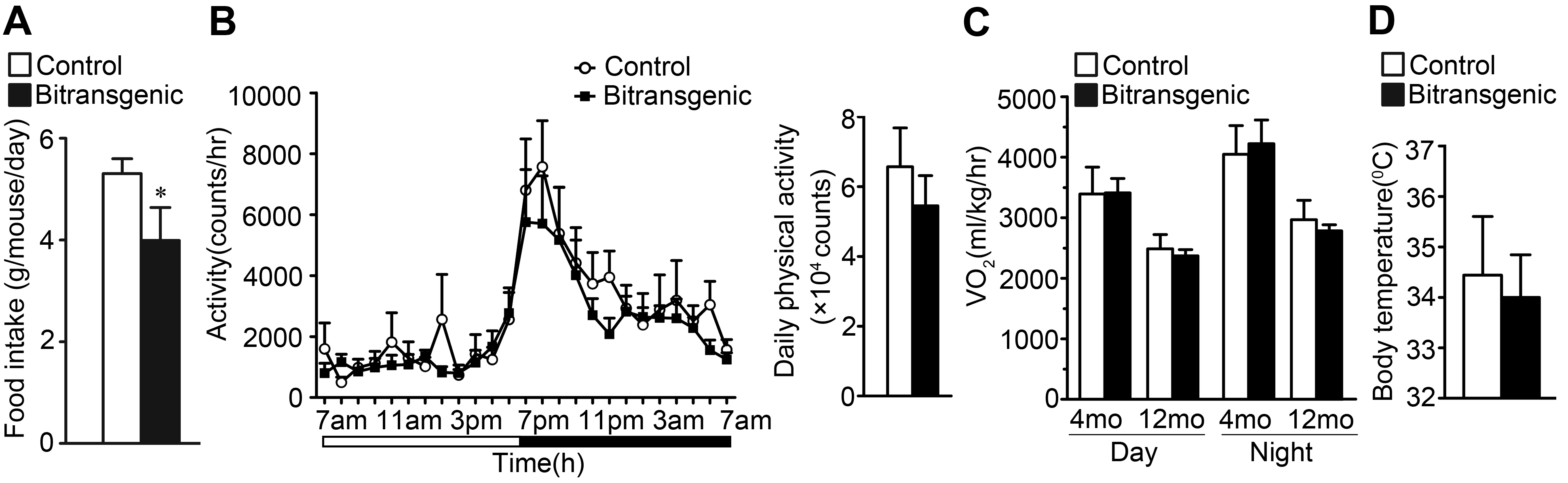

Supplement: Figure S3 — Male bitransgenic mice show similar physical activity, oxygen consumption, body temperature and decreased food intake. (A) Food intake of male bitransgenic mice decreased when compared with littermate controls (n = 5 for each group). (B) Physical activity did not change in male bitransgenic mice at 16 weeks of age, measured through a 12-h light/dark cycle (n = 5 for each group). Physical activity was presented as mean ± SEM. (C) Oxygen consumption did not change in male bitransgenic mice (n = 4–5 for each group). (D) Body temperature did not change in male bitransgenic mice at 8 months of age (n = 5 for each group). * P<0.05 versus littermate controls. (TIF) [file pone.0021759.s003.tif]

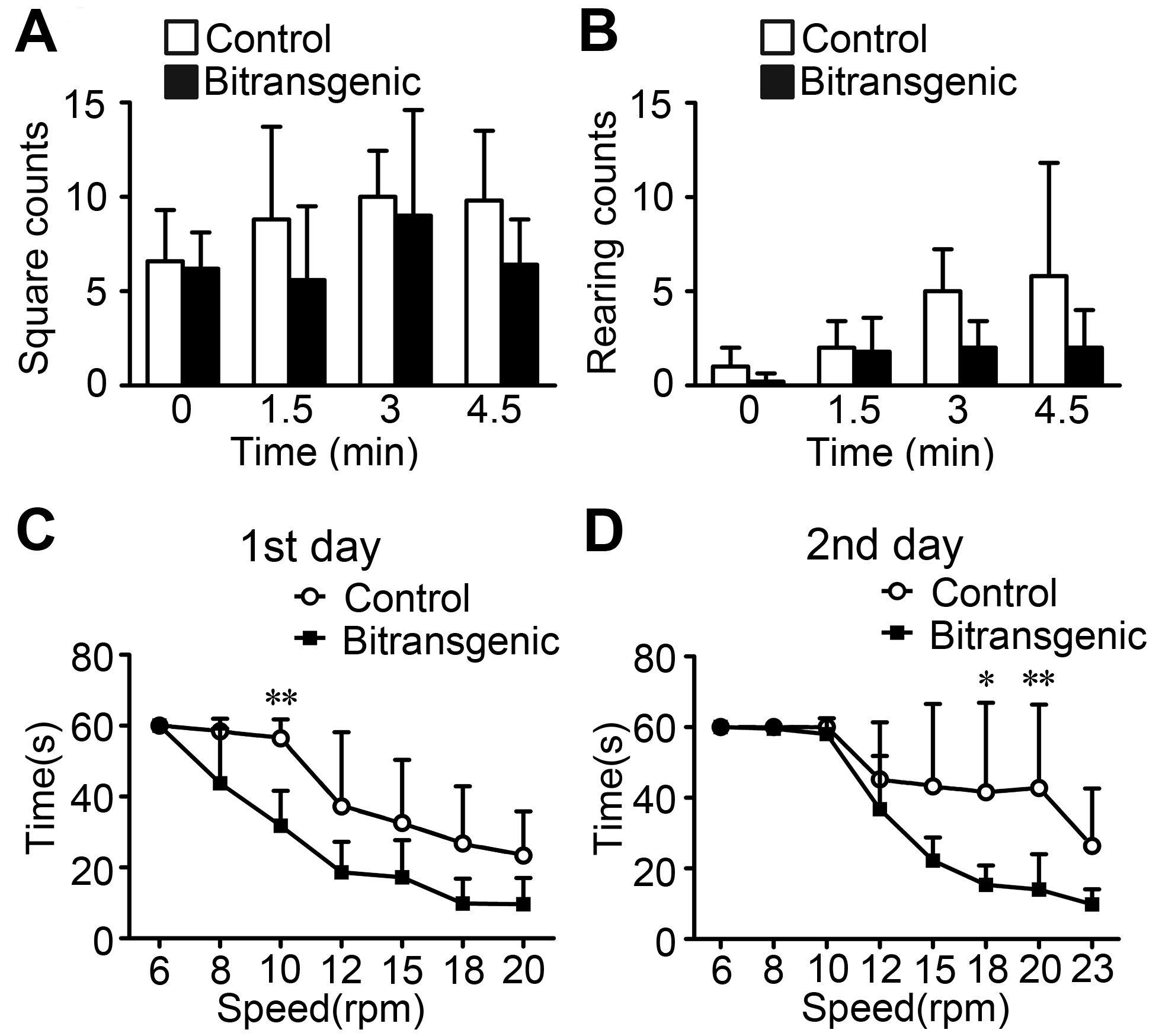

Supplement: Figure S4 — Male bitransgenic mice show decreased motor behavior by open field and rotarod performance tests. (A) Crossed squares in the open field test did not change in male bitransgenic mice (n = 5 for each group). Two-way ANOVA showed no significant difference between groups. (B) Male bitransgenic mice had similar number of rearings in the open field test (n = 5 for each group). Two-way ANOVA showed no significant difference between groups. (C) Rotarod performance on the first day was decreased in male bitransgenic mice (n = 5 for each group). ** P<0.01 versus littermate controls by two-way ANOVA. Two-way ANOVA showed significant difference between curves, P = 0.0225. (D) Rotarod performance on the second day was slightly decreased in male bitransgenic mice (n = 5 for each group). * P<0.05, ** P<0.01 versus littermate controls by two-way ANOVA. (TIF) [file pone.0021759.s004.tif]

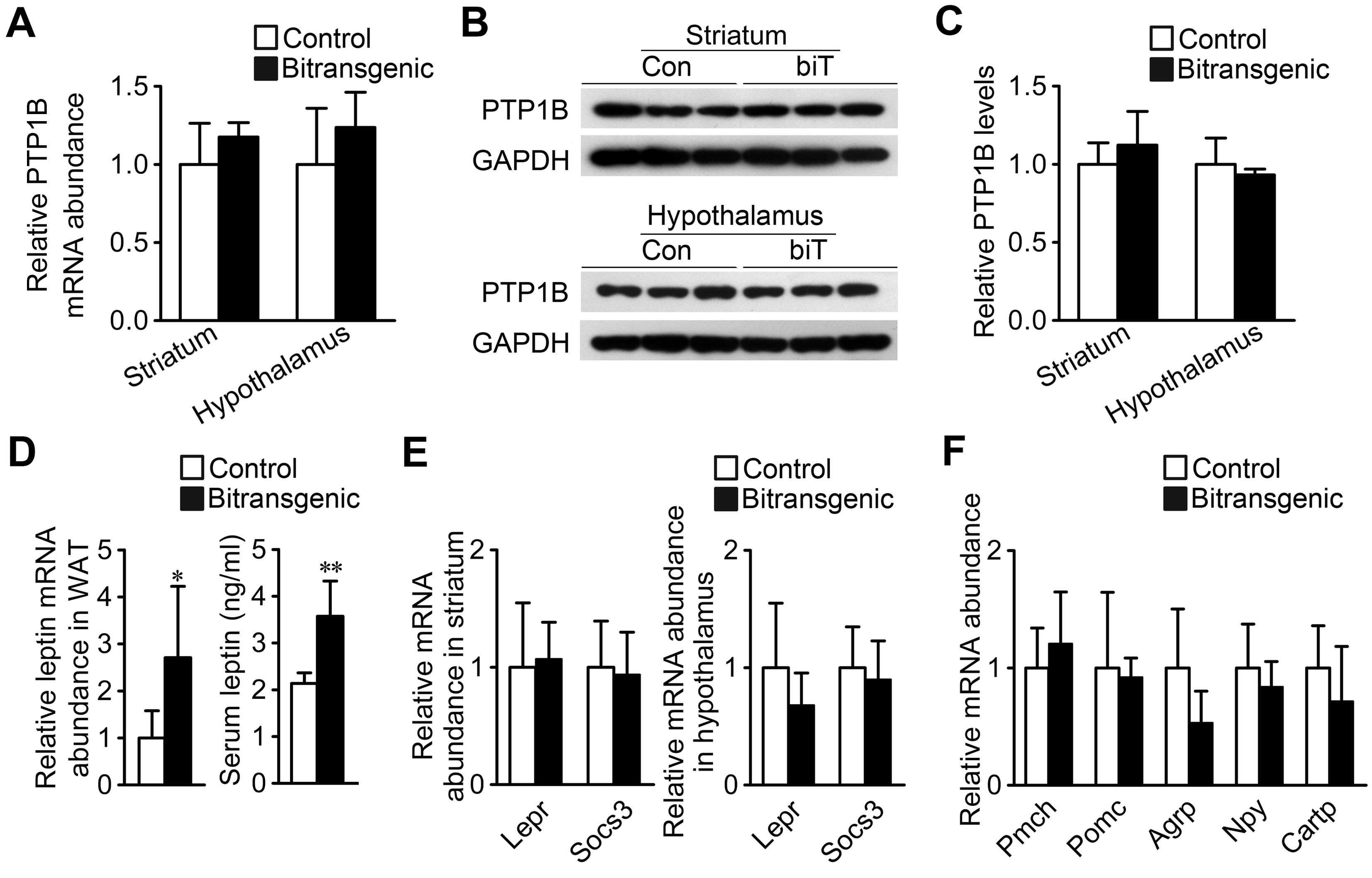

Supplement: Figure S5 — Leptin is upregulated in female bitransgenic mice, and the expression of PTP1B, Lepr, Socs3 and some feeding related neuropeptides is not changed in striatum or hypothalamus. (A) The mRNA levels of PTP1B in the striatum (n = 3 for each group) and hypothalamus (n = 6–7 for each group) of female bitransgenic mice were not changed. (B) The protein levels of PTP1B in the striatum and hypothalamus of female bitransgenic mice were not changed (n = 3 for each group). GAPDH was measured as loading control. (C) Quantification of the relative PTP1B protein levels corresponding to (B). (D) The mRNA levels of leptin in WAT (n = 3 for each group) and fed serum leptin levels (n = 10 for each group) of female bitransgenic mice were significantly elevated. (E) The mRNA levels of Lepr, Socs3 in the striatum (n = 3 for each group) and hypothalamus (n = 6–7 for each group) of female bitransgenic mice were not changed. (F) The mRNA levels of some feeding related neuropeptides in hypothalamus of female bitransgenic mice were not changed (n = 6–7 for each group). * P<0.05, ** P<0.01 versus littermate controls. (TIF) [file pone.0021759.s005.tif]

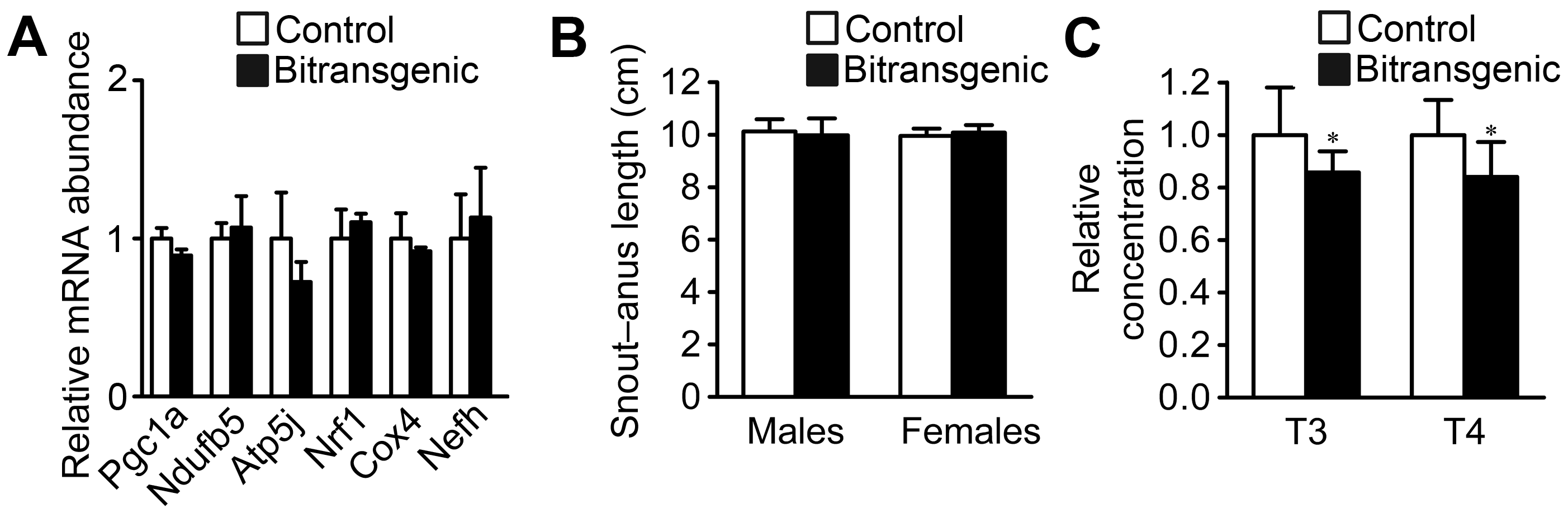

Supplement: Figure S6 — The mRNA levels of Pgc1a related genes in the striatum of female mice, the mouse snout-anus length and male serum T3, T4 levels. (A) The expression of Pgc1a and its related genes did not alter in the striatum of female bitransgenic mice (n = 3 for each group). (B) The mouse snout-anus length was similar between bitransgenic mice and controls (n = 6–7 for each group). (C) Serum T3 and T4 levels were decreased in male bitransgenic mice (n = 10–11 for each group). * P<0.05 versus littermate controls. (TIF) [file pone.0021759.s006.tif]

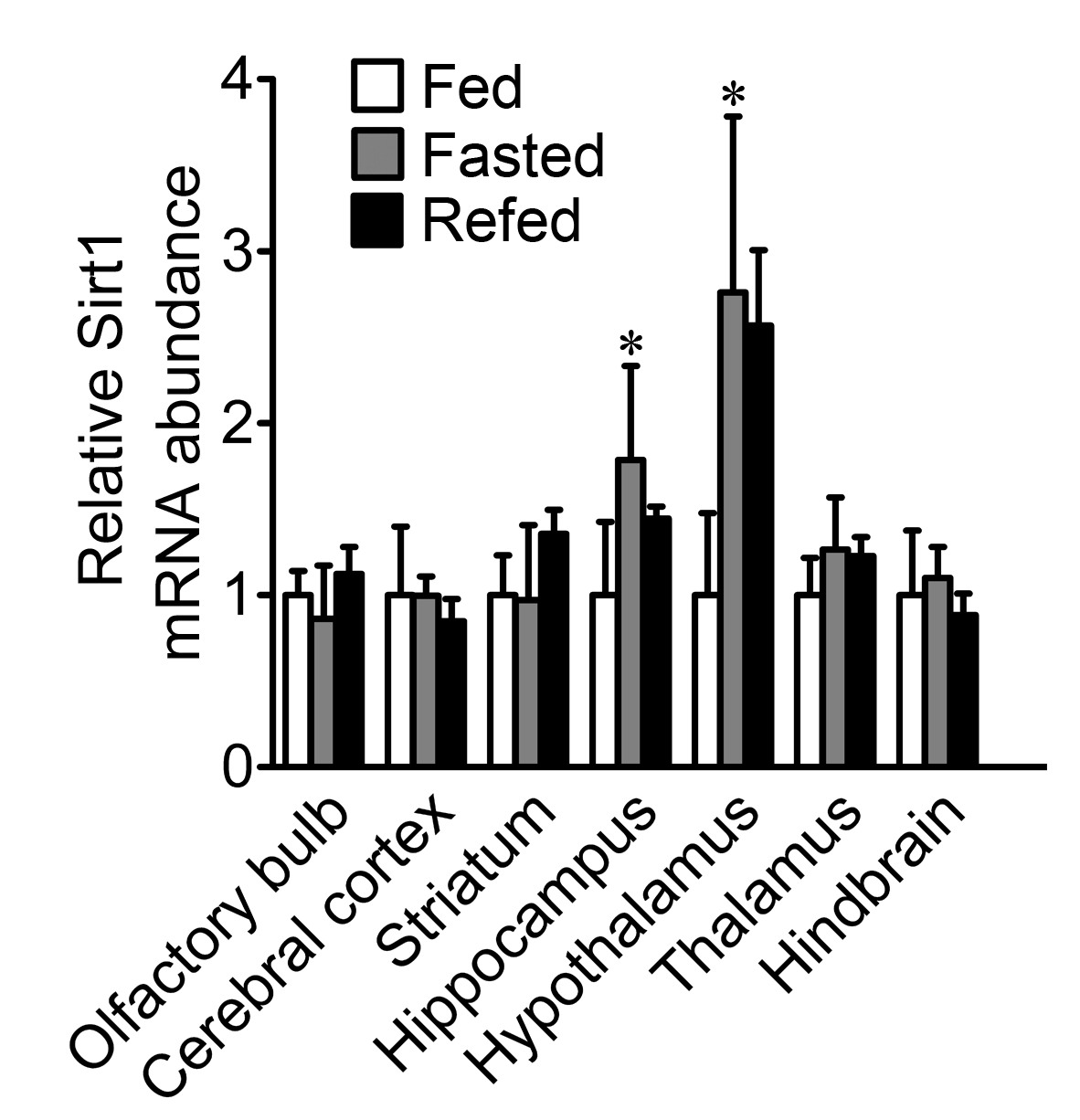

Supplement: Figure S7 — The Sirt1 mRNA levels in different brain regions under feeding, fasting and refeeding conditions. The Sirt1 mRNA levels of olfactory bulb, cerebral cortex, striatum, hippocampus, hypothalamus, thalamus and hindbrain from 8-week-old female mice fed ad libitum, fasted for 24 h, or fasted for 24 h and refed for 24 h were analyzed by real-time PCR (n = 5–7). * P<0.05 versus fed ad libitum. (TIF) [file pone.0021759.s007.tif]
