# Supplementary material for: Overexpression of SIRT1 in Mouse Forebrain Impairs Lipid/Glucose Metabolism and Motor Function
Source: PLoS One. 2011 Jun 30;6(6):e21759. doi: 10.1371/journal.pone.0021759 (PMC3128079; doi:10.1371/journal.pone.0021759)
Supplement: Table S1 — Primers used in real-time PCR. (DOC) [file pone.0021759.s008.doc]

**Supplemental Table 1: Primers used in real-time PCR.**

| Gene | 5’ primer | 3’ primer |
| --- | --- | --- |
| 36B4 | CACTGGTCTAGGACCCGAGAA | AGGGGGAGATGTTCAGCATGT |
| Acca | GTCCCCAGGGATGAACCAATA | GCCATGCTCAAGGAAAGTAGC |
| Fasn | AAGTTGCCCGAGTCAGAGAA | CGTCGAACTTGGAGAGATCC |
| Lpl | TGGAGAAGCCATCCGTGTG | TCATGCGAGCACTTCACCAG |
| Ap2 | ACACCGAGATTTCCTTCAAACTG | CCATCTAGGGTTATGATGCTCTTCA |
| Cd36 | ATGGGCTGTGATCGGAACTG | GTCTTCCCAATAAGCATGTCTCC |
| Pparg | GTGCCAGTTTCGATCCGTAGA | GGCCAGCATCGTGTAGATGA |
| Pparg1 | TTTAAAAACAAGACTACCCTTTACTGAAATT | AGAGGTCCACAGAGCTGATTC |
| Pparg2 | GATGCACTGCCTATGAGCACTT | AGAGGTCCACAGAGCTGATTC |
| Cebpa | GAACAGCAACGAGTACCGGGTA | GCCATGGCCTTGACCAAGGAG |
| Ldlr | GTTGACGGCTCCCATGAGTG | GTCCTTGCAGTCTGCCTCG |
| Srebf1 | GGAGCCATGGATTGCACATT | GGAAGTCACTGTCTTGGTTGTTGA |
| Gpam | ACGCACACAAGGCACAGAG | TGCTGCTCAGTACATTCTCAGTA |
| Dgat1 | TGTTCAGCTCAGACAGTGGTT | CCACCAGGATGCCATACTTGAT |
| Mlycd | CACTTCCGGCGCACCAA | CGAGCCTAGAACTTTCATCAGG |
| Cpt1b | CTCCTGGAAGAAACGCCTTATT | CACCTTGCAGTAGTTGGAACC |
| Acadm | AGTACCCGTTCCCTCTCATCA | CCATACGCCAACTCTTCGGTAA |
| Ppara | AGAGCCCCATCTGTCCTCTC | ACTGGTAGTCTGCAAAACCAAA |
| Glut4 | ACTCATTCTTGGACGGTTCCTC | CACCCCGAAGATGAGTGGG |
| G6pase | GAAAAAGCCAACGTATGGATTCC | CAGCAAGGTAGATCCGGGA |
| Pepck | CTGGCACCTCAGTGAAGACA | TCGATGCCTTCCCAGTAAAC |
| Pgc1a | CCAGCCTCTTTGCCCAGAT | GTCGCTACACCACTTCAATCCA |
| Pdk4 | ATCTAACATCGCCAGAATTAAACC | GGAACGTACACAATGTGGATTG |
| Mfn2 | ACGTCAAAGGGTACCTGTCCA | CAATCCCAGATGGCAGAACTT |
| Ucp1 | ACTGCCACACCTCCAGTCATT | CTTTGCCTCACTCAGGATTGG |
| Ucp2 | CCTACAGCGCCAGATGAGCT | GAGTCGTAGAGGCCAATGCG |
| Ucp3 | AGATGGTGGCTCAGGAGGG | CCCAGACGCAGAAAGGAGG |
| Cox8b | GAA CCA TGA AGC CAA CGA CT | GCGAAGTTCACAGTGGTTCC |
| Sirt1 | TTGGCACCGATCCTCGAAC | CCCAGCTCCAGTCAGAACTAT |
| Grin2a | AGCCCCCTTCGTCATCGTAGA | ACCCCTTGCAGCACTTCTTCAC |
| Grin2b | CTTAATCTGTCCGCCTAGAGCTTT | TGCGCTGGGCTTCATCTT |
| Cnr1 | TCCCACAGAAATTCCCTCTAACT | CCTTGAACGATGAGAGAGACTTG |
| Slc1a3 | TTACCTGTTTCGGAATGCCTTC | CCCGGTAGCTCATTTTATACGG |
| Slc18a2 | TCACCAACCCATTCATAGGACT | CAAGAGGAGCCGATTCCCTG |
| Dat | CCTGCTCTCAGTCATCGGC | CATCCCGGCAATAACCATGAA |
| Ddc | TACCCAGCTATGCTTGCAGAC | GCGGATAACTTTAGTCCGAGC |
| Ache | CTTCTGGAACCGCTTCCTCCCCAAATT | GTAGTGGTCGAACTGGTTCTTCCAGTGCAC |
| Gad1 | CACAGGTCACCCTCGATTTTT | ACCATCCAACGATCTCTCTCATC |
| [Ghrh](http://www.ncbi.nlm.nih.gov/gene/14601) | TGTATGCCCGGAAAGTGATCC | CTCCAGGGTCATCTGCTTGTC |
| Sst | ACCGGGAAACAGGAACTGG | TTGCTGGGTTCGAGTTGGC |
| [Gnrh1](http://www.ncbi.nlm.nih.gov/gene/14714) | AGCACTGGTCCTATGGGTTG | GGGGTTCTGCCATTTGATCCA |
| [Crh](http://www.ncbi.nlm.nih.gov/gene/12918) | CTGGTGGCTCTGTCGTCCTGC | TGCTCCGGCTGCAAGAAATTC |
| [Trh](http://www.ncbi.nlm.nih.gov/gene/22044) | CAGGGACCTTGGCTGATGATG | CCGGACCTGGACTTTCTCC |
| Ndufb5 | CAAGAGACTGTTTGTCGTCAAGC | TGTTCACCAGTGTTATGCCAAT |
| [Atp5j](http://www.ncbi.nlm.nih.gov/gene/11957) | TATTGGCCCAGAGTATCAGCA | GGGGTTTGTCGATGACTTCAAAT |
| Nrf1 | AGCACGGAGTGACCCAAAC | TGTACGTGGCTACATGGACCT |
| Cox4 | ATTGGCAAGAGAGCCATTTCTAC | CACGCCGATCAGCGTAAGT |
| Nefh | GTTCCGAGTGAGGTTGGACC | GCCGGTACTCAGTTATCTCCT |
| [Pmch](http://www.ncbi.nlm.nih.gov/gene/110312) | GTCTGGCTGTAAAACCTTACCTC | CCTGAGCATGTCAAAATCTCTCC |
| [Pomc](http://www.ncbi.nlm.nih.gov/gene/18976) | AAGATGCCGAGATTCTGCTACA | GGGCTGTTCATCTCCGTTG |
| [Agrp](http://www.ncbi.nlm.nih.gov/gene/11604) | GCTGTGTAAGGCTGCACGAG | TCCATTGGCTAGGTGCGACT |
| [Npy](http://www.ncbi.nlm.nih.gov/gene/109648) | TCCGCTCTGCGACACTACAT | GGCGTTTTCTGTGCTTTCC |
| [Cartpt](http://www.ncbi.nlm.nih.gov/gene/27220) | CCCGAGCCCTGGACATCTA | GCTTCGATCTGCAACATAGCG |
| Lep | CACCAGGCTCCCAAGAATCATGTA | GGGATGGCTCTTATCTCTACTTGCT |
| [Lepr](http://www.ncbi.nlm.nih.gov/gene/16847) | CCTCCAGGAGAGATGCTCACAC | TGACTGTGCGTGGAACAGGT |
| [Socs3](http://www.ncbi.nlm.nih.gov/gene/12702) | GCGAGAAGATTCCGCTGGTA | TACTGATCCAGGAACTCCCGA |
| [Ptpn1](http://www.ncbi.nlm.nih.gov/gene/19246) | TGGCCACAGCAAGAAGAAAA | GGAAAGGCAGGATCTCTCGA |
